# Biphasic exocytosis of herpesvirus from hippocampal neurons and mechanistic implication to membrane fusion

**DOI:** 10.1038/s41421-019-0134-6

**Published:** 2020-01-14

**Authors:** Yun-Tao Liu, Sakar Shivakoti, Fan Jia, Chang-Lu Tao, Bin Zhang, Fuqiang Xu, Pakming Lau, Guo-Qiang Bi, Z. Hong Zhou

**Affiliations:** 1 Center for Integrative Imaging, Hefei National Laboratory for Physical Sciences at the Microscale, and School of Life Sciences, University of Science and Technology of China, Hefei, Anhui 230026 China; 2 California NanoSystems Institute, University of California, Los Angeles (UCLA), Los Angeles, CA 90095-7227 USA; 3 Department of Microbiology, Immunology and Molecular Genetics, UCLA, Los Angeles, CA 90095-7364 USA; 4 State Key Laboratory of Magnetic Resonance and Atomic and Molecular Physics, Key Laboratory of Magnetic Resonance in Biological Systems, Brain Research Center, Wuhan Institute of Physics and Mathematics, Chinese Academy of Sciences, Wuhan, 430071 China; 5 University of the Chinese Academy of Sciences, Beijing, 100049 China; 6 CAS Key Laboratory of Brain Function and Disease, and School of Life Sciences, University of Science and Technology of China, Hefei, Anhui 230026 China; 7 Center for Excellence in Brain Science and Intelligence Technology, Chinese Academy of Sciences, Shanghai, 200031 China

**Keywords:** Exocytosis, Membrane fusion, Cryoelectron tomography

## Abstract

Exocytosis is a crucial cellular process involved in the release of neural transmitters or signaling hormones, and disposal of waste or toxic materials. The relationship between structural transition and temporal progression of this process is poorly understood, partly due to lack of adequate tools to resolve such dynamic structures at sufficient resolution in 3D. Exocytosis can be hijacked by some viruses, exemplified by the widely used model α-herpesvirus pseudorabies virus (PRV), which relies on exocytosis for trans-synaptic spread across neurons. Here, we have used cryo electron tomography (cryoET) to capture 199 events of PRV exocytosis from cultured hippocampal neurons. We established cumulative frequency analysis to estimate the relative duration of an exocytosis stage based on the frequency of observed viral particles at that stage. This analysis revealed that PRV exocytosis is biphasic, including a fast, “release phase” driven by fusion proteins and fused membranes, and a slow, “recovery phase” driven by flattening of curved membranes. The biphasic property of exocytosis discovered here appears to be conserved for membrane fusion during viral entry, and our approach of cumulative frequency analysis should have general utility for characterizing other membrane fusion events.

## Introduction

Exocytosis is a cellular process by which cells use secretory vesicles to transport bulk materials onto the plasma membrane or out of the cell. It occurs during release of neural transmitters and signaling hormones^1^, disposal of waste or toxic materials^2^, as well as repair of membrane wounds^3,4^. Despite its fundamental significance in biology, the detailed mechanism underlying exocytosis is not well characterized due to the dynamic nature of this complex process, and the lack of adequate tools to resolve the three-dimensional, pleomorphic membrane-containing structures involved in its various transient steps. For example, synaptic exocytosis in neurons involves many specialized regulatory molecules and various mechanisms such as “full collapse” and “kiss and run”^5^. Optical reporters such as styryl dyes^6,7^, pHluorin (pH-sensitive green fluorescent protein)^8,9^, or quantum dots^10^ have been used to study the kinetics of exocytosis; however, the limited spatiotemporal resolution and signal-to-noise (S/N) ratio have hindered our ability to report exocytosis with details on structural change of membrane along with temporal progression.

PRV, a neurotropic α-herpesvirus, hijacks neuronal exocytosis mechanism to accomplish trans-synaptic spread, and has been widely used as a self-amplifying “live” tracer for the study of brain circuitry^11–13^. Trans-synaptic transport of virus can result in infection of higher order neurons or even viral encephalitis^14^. PRV particles travel to release sites inside acidified secretory vesicles containing various Rab GTPases^15^ and SNARE proteins^16^, both of which are key regulators of the plasma membrane-directed secretory pathway. Although it has been shown by fluorescence microscopy that exocytosis of the PRV involves vesicle docking, membrane fusion, and virion movement^15^, the structural details of these exocytosis steps remain elusive. Ease of access to numerous exocytosis events at transient stages in PRV-infected hippocampal neuron makes PRV-infected neurons an excellent model to study the dynamic mechanisms of vesicular exocytosis.

Cells infected with herpesvirus produce not only infectious virus particle but also noninfectious L-particles devoid of capsid and viral genome. L-particles can be formed independently to the virions and are also shown to facilitate the infection of herpesvirus in cell culture most likely by delivering additional tegument proteins to the target cell cytosol that is needed during viral replication^17,18^. Virion and L-particle formation occur in close proximity suggesting shared assembly and exit pathways, and use same constitutive secretory mechanism for exocytosis^19,20^. However, the release dynamics of PRV virion and L-particle in polarized cells like hippocampal neuron have not been studied in detail.

Cryo electron tomography (cryoET) overcomes these problems by plunge-freezing the cellular samples in liquid ethane, preserving the cell and the virus conditions in their near-native state. Moreover, rapid vitrification allows us to trap the viruses at different stages in the release process. With electron tomography, high-quality three-dimensional structures can be obtained, avoiding the possible overlapping of the cellular structure^21^.

In this study, we infected low-density cultures of hippocampal neurons at 6−7 days in vitro (DIV) with sparsely growing axons and dendrites, ideal for studying anterograde transport and the release of virus^21^. We used cryoET to obtain multiple snapshots of the neuronal exocytosis in situ to recapitulate the temporal progression of various transient steps of the entire exocytosis process by cumulative frequency analysis (CFA). We discovered a biphasic property of exocytosis, including a fast release phase and a slow recovery phase.

## Results

### Identification of virus exocytosis with cryoET

**Fig. 1:**
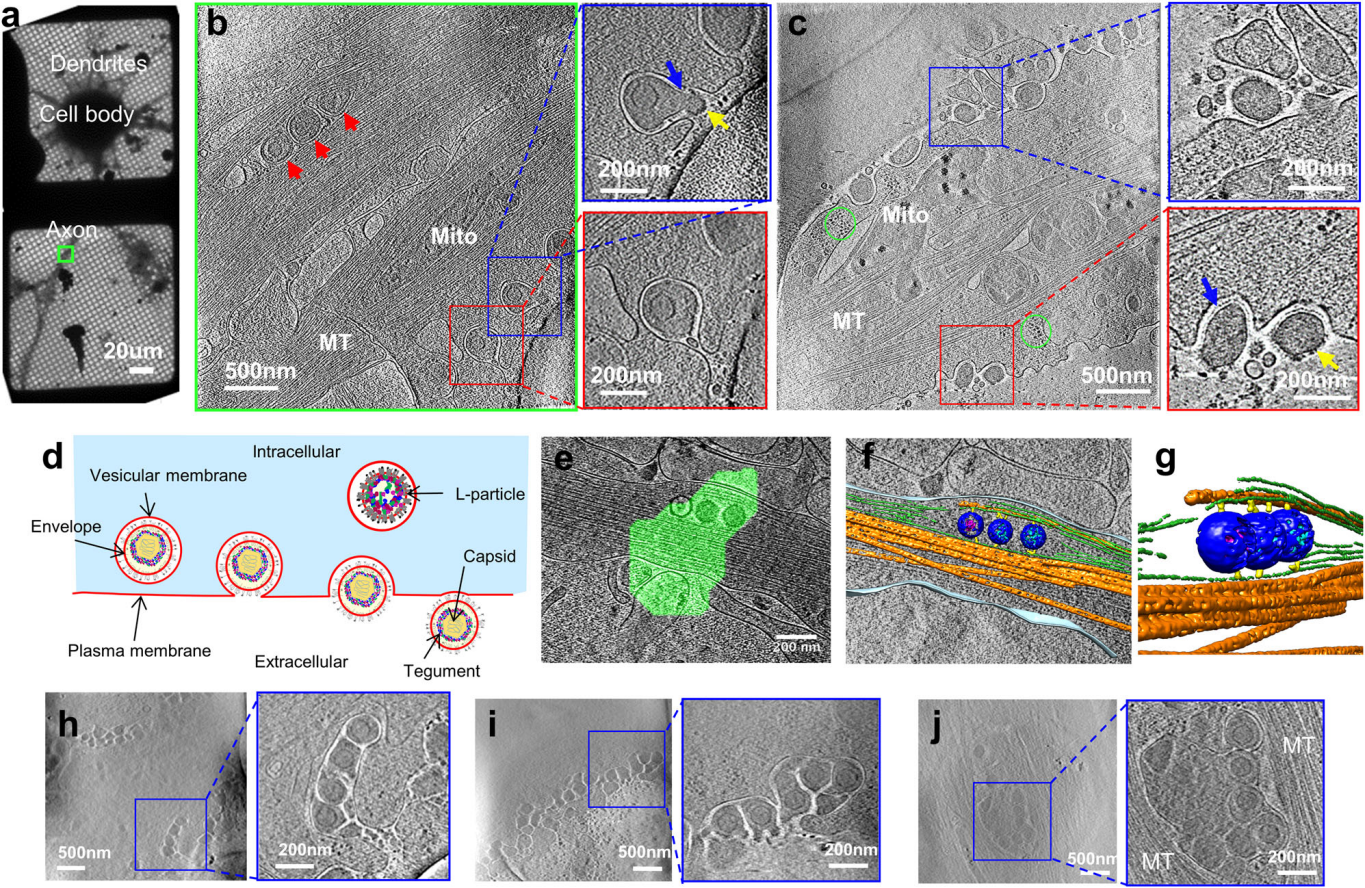
CryoET of cultured hippocampal neurons infected by PRV and capturing of progeny virus exocytosis. **a** Overview of a hippocampal neuron grown on a gold cryoEM grid. **b** Slices from the reconstructed tomogram of the area boxed in green of (**a**) showing single-particle exocytosis of viruses in axon. **c** Slices from the tomogram showing single-particle exocytosis of L-particles in dendrite. **d** Illustration of the PRV exocytosis process. **e** Composite images of the fluorescent signal from PRV-Bartha capsids during retrograde transport. **f**, **g** Segmentation of the region shown in (**e**) are capsids (blue), viral genome (cyan), scaffolding proteins (magenta), microtubules (orange), other filament structures (green) and densities connecting capsid and filaments (yellow), which are either overlaid on a slice (gray) from the tomogram (**f**) or blown up (**g**) after a 90° rotation from the view of (**f**). **h**–**j** Slices from the tomogram showing multiple-particle exocytosis in a “pea-pod”-like (**h**) and “marble-pouch”-like (**i**) release cavity, and enveloped viruses or L-particles being transported in a vesicle (**j**). Insets next to panels (**b**, **c** and **h**−**j**) are zoom-in views of their boxed areas. Red arrowhead: enveloped virus inside secretory vesicle during transport, yellow arrowhead: glycoprotein spikes, blue arrowhead: protein density between envelope and vesicular membrane, green circles: polyribosomes. Mito mitochondria, MT microtubules.

The hippocampus is a typical target of infection in herpesvirus encephalitis in human^22^. To gain insights into the essential steps of viral exocytosis, we cultured hippocampal neurons on gold EM grids and then infected the cells with PRV (Becker strain). The neurons cultured for 6 DIV contained axons and dendrites thin enough for direct observation by cryo electron microscopy (cryoEM), and we did not observe synaptic contact in such low-density culture. We identify putative axons as long neuronal processes (Fig. 1a), which contain parallel microtubules but lack rough endoplasmic reticulum (Fig. 1b). By contrast, putative dendrites taper at the end (Fig. 1a), have fewer compacted microtubules, and contain ribosomes (Fig. 1c).

At 14–16 h post infection (hpi), viral particles began to show up in vesicles interacting with plasma membrane (Fig. 1a–d). These particles were interpreted as progeny viral particles in the process of exocytosis for the following reasons: First, enveloped viruses were observed inside cellular secretory vesicles (Supplementary Fig. S1a), but PRV is known to enter the neuronal cells by direct fusion with the plasma membrane and not by transcytosis or endocytosis^23,24^. Second, neurons on grids infected with PRV were frozen at 14–16 hpi, which is the time established by others as the ideal time to observe progeny PRV transport and exocytosis^15,25,26^. Third, the neurons in our culture were at low density and were well separated from one another. We acquired our cryoET tilt series in places only containing single axon or dendrite (e.g., Fig. 1a), thus preventing input viruses from getting “squeezed” between axons. To further confirm that the observed virions were progeny virus particles undergoing exocytosis during egress rather than endocytosis during entry, we used cryo correlative fluorescence and electron microscopy (cryoCLEM) to image neurons infected by PRV Bartha, a strain mainly capable of retrograde transport (thus rarely producing viral particles in secretory vesicles in axon). Indeed, through our study, we only observed naked capsids, attaching to cytoskeletons for retrograde transport (Fig. 1e–g; Supplementary Fig. 1b and Supplementary Movie 1).

### Single- and multiple-particle exocytosis

We have obtained a total of 96 cryoET tomograms of neurons infected by PRV Becker strain. Careful mining of the 3D structures identified two distinct types of viral particles-containing vesicles interacting with plasma membrane. The first, dominating type contains one viral particle, representing vesicles undergoing what we termed single-particle exocytosis (Fig. 1b, c). The second type contains multiple particles (Fig. 1h, i), which we interpreted to be instances undergoing multiple-particle exocytosis, because PRV does not enter in neurons by endocytosis^23,24^. From our tomograms, we observed 199 single-particle exocytosis events and only 20 multiple-particle exocytosis events. During single-particle exocytosis, the vesicular membrane enclosing a single viral particle fuses with the plasma membrane, forming a cavity and a pore. The viral envelope facing the vesicular membrane inside the cavity is lined by a distinct protein density (blue arrows in Fig. 1b, c), while that exposed to the pore is decorated by glycoprotein spikes (yellow arrows in Fig. 1b, c). Intriguingly, we observed two unique scenarios of multiple-particle exocytosis. In the first scenario, viruses or L-particles lined up at the release pore, making a “pea-pod”-like release cavity (Fig. 1h). In the second scenario, multiple viral particles were undergoing exocytosis in a “marble pouch”-like release cavity (Fig. 1i). Such secretory vesicles enclosing multiple viral particles were observed during transport (Fig. 1j). We suggest that the first and second scenarios of multiple-particle exocytosis are caused by vesicles fusing with the release cavity in tandem and by single vesicle that encloses multiple particles fusing with plasma membrane, respectively.

### Estimating relative elapse time of exocytosis by cumulative frequency analysis

**Fig. 2:**
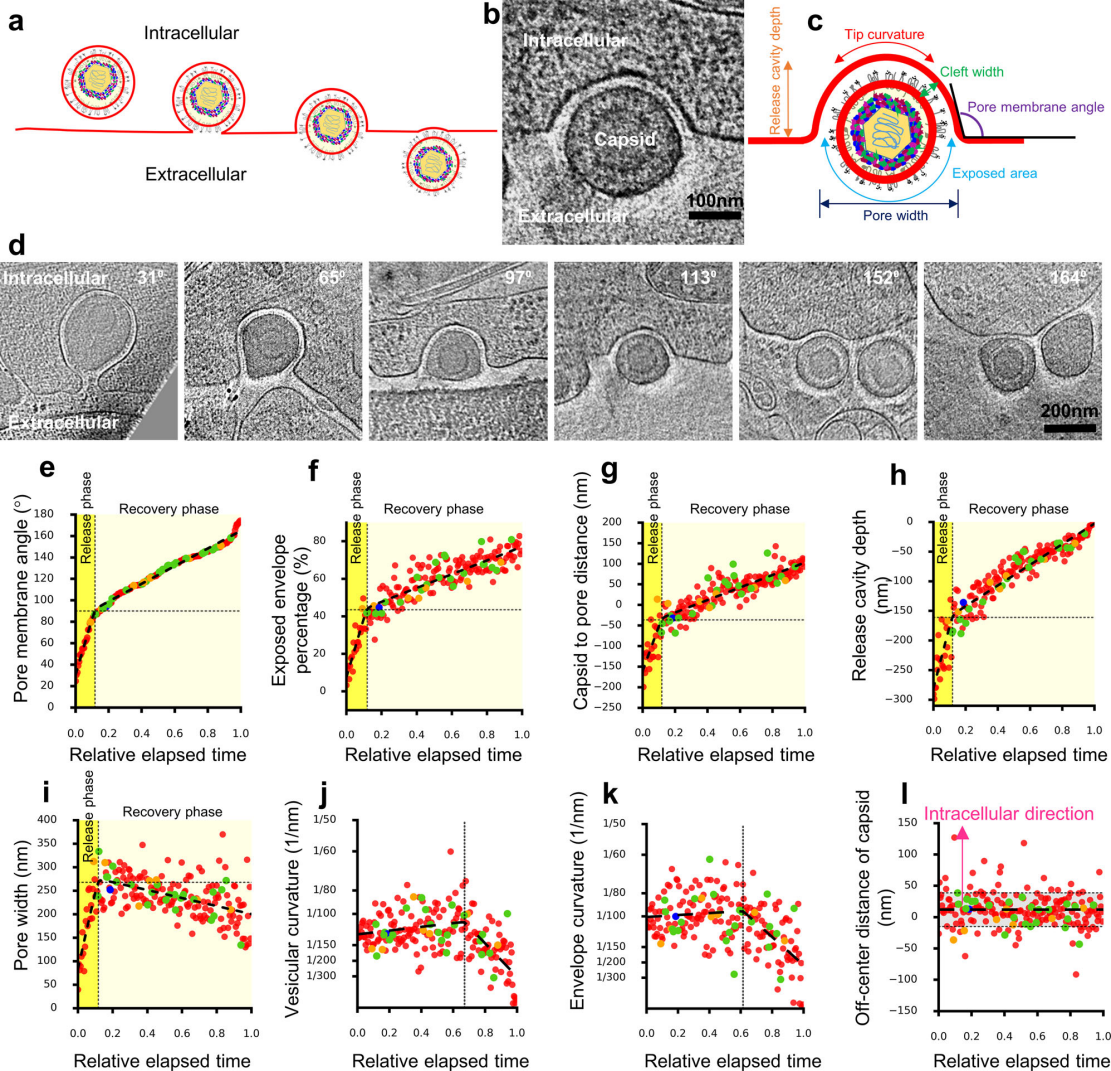
Discovery of two phases of single-particle exocytosis of PRV. **a** Cartoon representation of single-particle exocytosis. **b**, **c** Tomographic slice (**b**) and its corresponding schematic illustration depicting exocytosis parameters (**c**). **d** Representative snapshots from tomograms of single-particle exocytosis characterized by the increasing pore membrane angle (upper right of each image) from the point of fusion pore formation to complete release of progeny viruses. **e**–**l** Scattered plots showing pore membrane angle (**e**), percentage of the exposed area versus total surface area of envelope (**f**), distance from center of capsid to the plane of the release pore (**g**), release cavity depth (**h**), pore width (**i**), vesicular curvature (**j**), envelope curvature (**k**), and distance from center of capsid to the center of envelope (**l**) plotted as a function of relative elapsed time of single-particle exocytosis. In (**e**–**k**), a hyperbola (black dashed lines) is fitted to the data points. Vertical dotted lines in (**e**–**i**) show the point of transition separating the exocytosis into a release and recovery phase, whereas in (**j** and **k**), represent the timepoint when the membrane flattening initiated. Horizontal dotted line shows value of each parameters at phase transition point. In (**l**), negative and positive values represent the center of capsid outward and inward from the center of the envelope, respectively. Horizontal dashed line and gray area indicate the mean and standard deviation (SD), respectively. Dots are colored to differentiate the number of clathrin-coated pits (CCPs) invaginating from vesicular membrane during the single-particle exocytosis: one (green), two (yellow), four (blue), or without (red) CCPs.

Viral exocytosis can be divided into multiple stages, beginning with the attachment of a secretory vesicle to the cytoplasmic membrane and fusion pore formation (time 0), through widening of the pore, reaching a maximal pore opening, followed by membrane flattening/pore narrowing, and ending with a progeny virus escaping from the membrane (final stage) (Fig. 2a). Pore membrane angle, defined as the angle between the two membranes at the junction of plasma and vesicular membranes (Fig. 2b, c), supplementary to invagination angle defined by Avinoam et al.^27^, is an index independent of vesicle sizes and increases monotonically from beginning to the end during exocytosis. Thus, it was selected as a surrogate to chronologically separate the stages of virus exocytosis. We consider virus exocytosis event with the minimum pore membrane angle as the beginning of exocytosis and that with the maximum pore membrane angle as the end of exocytosis.

In cryoET, we can only obtain 3D snapshots of many viral particles at different stages of exocytosis (Fig. 2d), rather than tracing one particular viral particle going through all these stages. However, we can estimate the duration of a viral particle at a particular stage from the frequency of the observed viral particles at that stage. We define relative elapsed time as the elapsed time for viral exocytosis from minimum pore membrane angle (time 0*)* to the particular stage of pore membrane angle divided by the total elapsed time of virus exocytosis. Thus, the relative elapsed time can be calculated as the cumulative frequency of viral particles with a specific pore membrane angle.

### Discovery of biphasic viral exocytosis

Scatter graph of the pore membrane angle as a function of the relative elapsed time of single-particle exocytosis showing PRV Becker exocytosis is characterized by two linear phases with distinctive slopes: the first steep and the second gradual (Fig. 2e). To quantitatively characterize the two phases, we fitted the scattered plot with a hyperbola curve, which has two (linear) asymptotes and a vertex (Supplementary Fig. S2a–d). Thus, the slopes of the two asymptotes of the fitted hyperbola curve represent the rates of increase of the pore membrane angle in the two phases, and the relative elapse time at the hyperbolic vertex represents the phase transition time (Fig. 2e). The two phases can also be verified by other parameters including exposed envelope percentage (Fig. 2f), the distance from center of capsid (Fig. 2g) or envelope (Supplementary Fig. S2e) to release pore, and depth of release cavity (Fig. 2h). When these parameters are plotted as functions of the relative elapsed time, they can also be well fitted with hyperbola curves whose vertices correspond to the phase transition time.

In the first phase, fusion pore widens (Fig. 2i), instigating viral release. By contrast, in the second phase, fusion pore narrows and plasma membrane starts to recover (Fig. 2i). Thus, we named the first and second phases as release phase and recovery phase, respectively. The duration of the release phase is shorter, being only one-seventh of that of the recovery phase (Fig. 2e). Correspondingly, capsid moved 7.2 times faster in the release phase (Fig. 2g). At the end of the recovery phase, a steep slope can be observed in the cumulative frequency plot (Fig. 2e), suggesting that the virus moves faster to detach the cell.

Three sources of energy could drive the exocytosis: first, energy-level differences of the fusion proteins between their meta-stable, higher energy prefusion state and stable, lower-energy postfusion state; second, membrane tension between the fused vesicular and plasma membranes (Fig. 2c); third, energy derived from the flattening of the vesicular membrane (Fig. 2c). The continuously increasing pore membrane angle (Fig. 2e) indicates that membrane tension between the fused vesicular and plasma membranes (i.e., the second source of energy) reduced throughout the exocytosis process. The release phase probably draws energy from the first two sources. As the first source of energy is depleted in the release phase, exocytosis slows down and enters the recovery phase. Our data show that the initiation of the flattening of the vesicular membrane (and viral envelope) takes place only at the later stage of the recovery phase (Fig. 2j, k), suggesting the energy released from flattening of the vesicular curvature (i.e., the third source of energy) only contributes to the later stage of the recovery phase.

Previous studies showed that the nucleocapsid occupied an eccentric position inside the envelope of herpesviruses^23,28^. We observed that ~20% of the envelope area remained tethered to the plasma membrane for the virions at the end of the recovery phase, resulting in a flattened envelope on the lagging edge of the released virion (Fig. 2d last panel, f, k). Throughout the entire exocytosis process, the maximal depth of the release cavity was ~300 nm, corresponding to the size of secretory vesicle enclosing a PRV virion. Cleft width (Supplementary Fig. S2f), envelope radius (Supplementary Fig. S2g), and capsid-envelope eccentricity (Fig. 2l) stayed the same during exocytosis. Moreover, most of the capsids (71%) were located near the lagging edge (off-center distance > 0) of the virion during exocytosis (Fig. 2l). Thus, though not always, vesicular membrane in contact with capsid-distal edge of the virus envelope fused with target cell membrane during exocytosis in a statistically significant way (*P* < 0.01 by *t* test).

### Clathrin-coated pits were observed during single-particle exocytosis

**Fig. 3:**
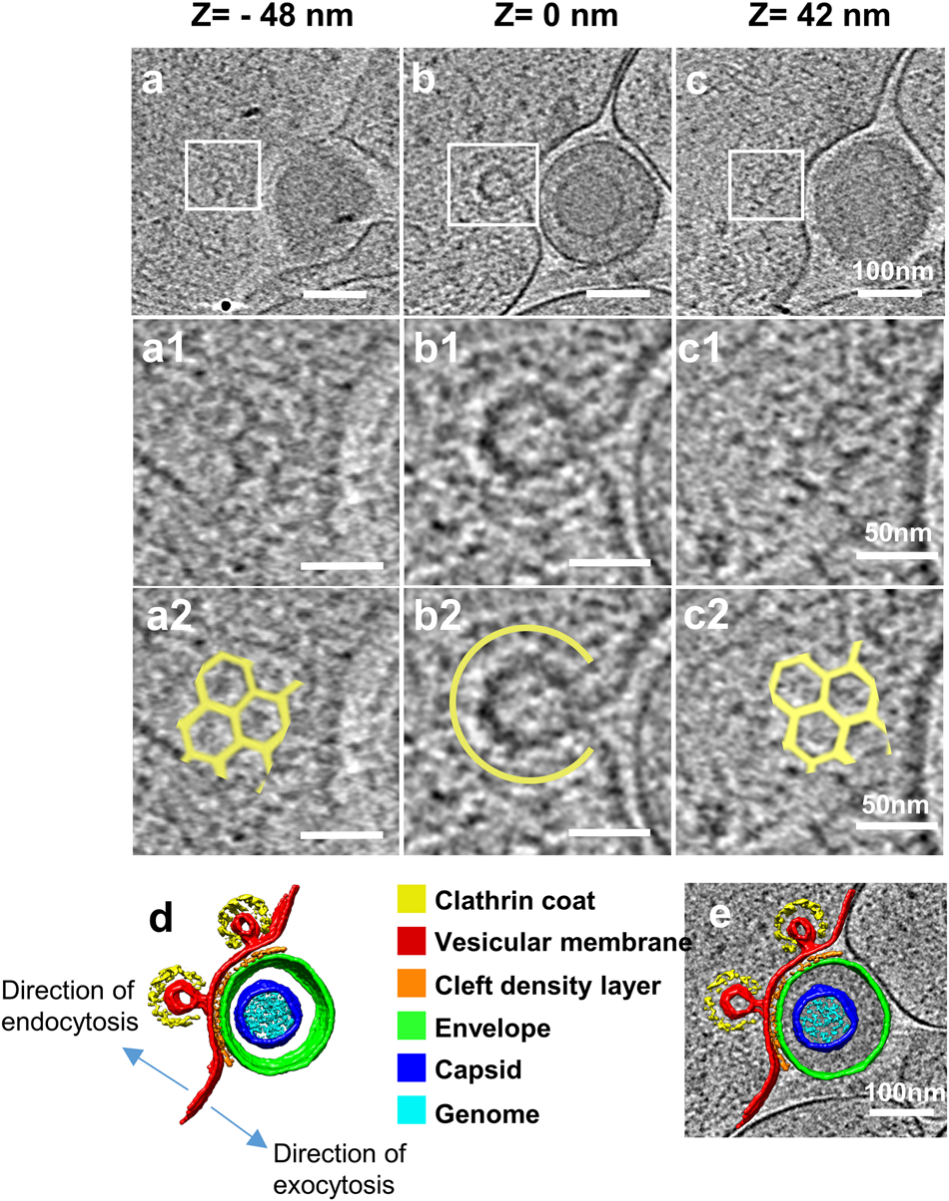
Virus exocytosis involves clathrin-coated pit (CCP). **a**–**c** Three 4.5-nm-thick slices extracted from a tomogram 48 nm below (**a**), exactly at (**b**), and 42 nm above (**c**) the central plane of the virus during exocytosis. **a1**–**c1** Zoom-in views of the boxed area showing clathrin lattices (**a1** and **c1**) and clathrin-coated pit (**b1**). **a2**–**c2** Schematic drawing of characteristic triskelion lattice (**a2** and **c2**) and clathrin coat (**b2**) superposed to (**a1**–**c1**). **d**, **e** 3D surface rendering of tomogram of a single-particle exocytosis with two clathrin-coated pits (**d**), superposed to central slice (**e**).

Intriguingly, 31 out of the 199 single-particle exocytosis events (~16%) were accompanied by invagination(s) on the vesicular membrane towards the cytosol (Fig. 3). Each invagination pit was coated by characteristic pentagonal and hexagonal densities (Fig. 3a–c; Supplementary Movie 2). The mean distance between the adjacent vertices of these polygons was 17 ± 0.4 nm, similar to the distance between adjacent triskelion vertices on clathrin coats^29,30^, suggesting that the invagination pits are coated by clathrin. The mean radius of the putative clathrin coat and that of the invagination pit was 46.3 ± 6.1 and 38.1 ± 6.7 nm, respectively. Among the 31 exocytosis events with the clathrin-coated pits (CCPs) we examined, 23 had one CCP, 7 had two CCPs and 1 had four CCPs (Figs. 2e–i, 3; Supplementary Fig. S3). We hypothesized that clathrin-mediated endocytosis might facilitate single-particle exocytosis by reducing the area of contact between the vesicular membrane and the envelope, which could also provide additional energy driving virus exocytosis. Because exocytosis and endocytosis are physiologically colocalized to certain “hot-spots” on a neuron^31,32^, the observation of CCP during exocytosis supports the notion that virions hijack such cellular “hot-spots” for their spread.

### Exocytosis of L-particle

**Fig. 4:**
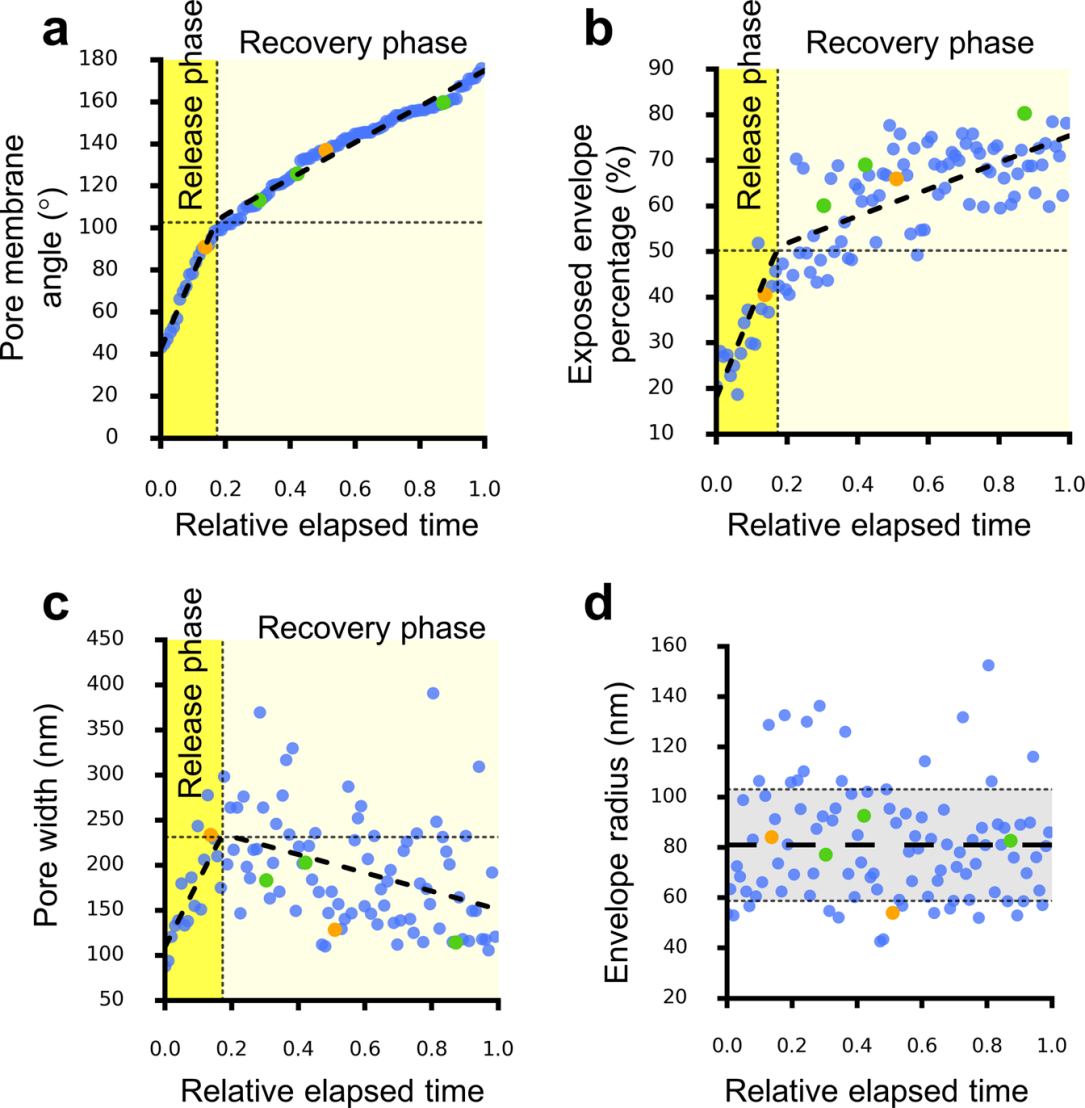
Exocytosis of L-particle. **a**–**d** Pore membrane angle (**a**), percentage of exposed area versus total area of envelope (**b**), release pore width (**c**), and envelope radius (**d**) plotted as the function of relative elapsed time of single-particle exocytosis of L-particle. In (**a**–**c**), a hyperbola (black dashed lines) is fitted to the data points. Vertical dotted lines in (**a**–**c**) show the point of transition separating the exocytosis into a release and a recovery phase. Horizontal dotted line in (**a**–**c**) shows value of each parameter at the phase transition point. In (**d**), horizontal dashed line and gray area indicate the mean and standard deviation (SD), respectively. Dots are colored to differentiate the number of clathrin-coated pits (CCPs) invaginating from vesicular membrane during the exocytosis with one (green), two (yellow), or without (blue) CCPs.

We observed 102 cases of the single-particle exocytosis of L-particles, five of which were accompanied by CCPs on their vesicular membrane. This single-particle exocytosis of L-particle also exhibited distinct release and recovery phases as measured by the same parameters defined above, including pore membrane angle (Fig. 4a), exposed envelope percentage (Fig. 4b), release cavity depth (Supplementary Fig. S4a), distance from center of L-particle to the fusion pore (Supplementary Fig. S4b), and width of the fusion pore (Fig. 4c). Cleft width and envelope radius remained constant during the single-particle exocytosis of L-particle (Fig. 4d; Supplementary Fig. S4c). The measured cleft width of L-particles exocytosis (Supplementary Fig. S4d) was the same as that for virion exocytosis. However, the relative elapsed time of the release phase for L-particles exocytosis was 48.7% larger than that for virion exocytosis (Fig. 4a). In L-particle exocytosis, the ratio of L-particle movement speed between the release phase and the recovery phase is 5.7. This ratio is smaller than that of virion movement speed between the two phases of virion exocytosis (which is 7.2).

## Discussion

**Fig. 5:**
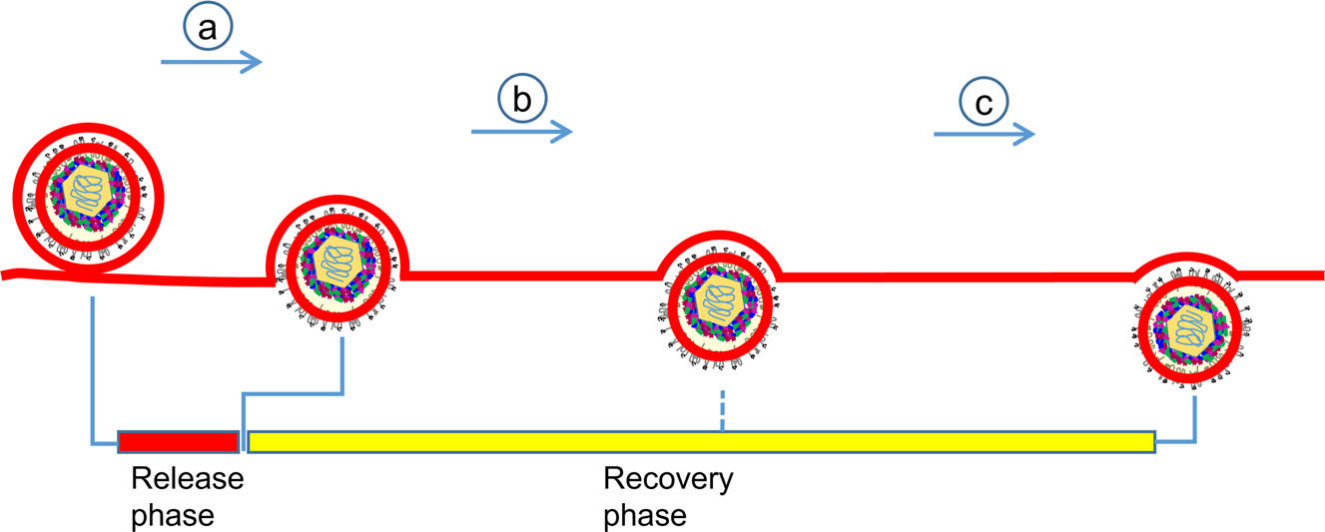
Schematic representation of single-particle exocytosis of PRV. **a** Release phase: The initial 11.7% (17.4% for L-particle) of the total single-particle exocytosis period where secretory vesicle enclosing virion attaches and fuses to the plasma membrane leading to the opening of fusion pore, and reaching maximal pore widening up to ~270 nm (~230 nm for L-particle). In this step, capsid moves ~100 nm outwards, exposing up to ~40% of envelope (~50% for L-particle) to extracellular space with marked reduction on release cavity from ~300 to ~150 nm (~200 to ~120 nm for L-particle). **b**, **c** Recovery phase: The following 89.3% (82.6% for L-particle) of the total single-particle exocytosis from maximal pore widening to the end of the exocytosis. In this step, capsid moves another ~100 nm outwards, exposing up to ~80% of envelope to extracellular space with a marked reduction on release cavity from ~150 to ~0 nm (~120 to 0 nm for L-particle). In (**b**), curvature of vesicular membrane and envelope stay the same, while in (**c**), curvature of vesicular membrane and envelope drops. Dashed line on the recovery phase indicates the initiation of envelope or vesicular membrane flattening.

Membrane fusion during exocytosis is mediated by a universal machinery that includes SNARE and “Sec1/Munc18-like” (SM) proteins^33^. Fusion energy generated by assembly of trans-SNARE complex forces the two membranes to interact^1^. SM proteins likely wrap around assembling trans-SNARE complexes to catalyze membrane fusion^1^. This membrane fusion step and the subsequent exocytosis step are rarely investigated structurally due to lack of adequate tools. Our approach of combining cryoET and cumulative frequency analyses has allowed the visualization of exocytosis and the understanding of its temporal progression. We discovered a fast release phase and a slow recovery phase during virion exocytosis (Fig. 5). Furthermore, during the recovery phase, secretory vesicles with CCPs were occasionally observed, similar to the occurrence of CCPs after synaptic vesicle exocytosis^9,34^. Thus, it is possible that viral exocytosis and synaptic exocytosis not only have conserved protein machinery for fusion, but also share common biophysical mechanisms to drive membrane dynamics.

The discovery of biphasic exocytosis offers a means to better characterize various cellular exocytosis events. For example, the increasing speed of pore membrane angle in the release phase was found to be seven times faster than that in the recovery phase. The ratio of release speed to the recovery speed, designated here as the exocytosis coefficient for convenience of discussion, can be calculated to characterize different types of exocytosis. A quick release phase is vital for fast withdrawal of vesicular content, followed by a slower recovery phase of membrane recuperation. The speed for recovery phase driven by membrane-flattening is likely the same for different kind of exocytosis; therefore, exocytosis coefficient reflects the efficiency of vesicle release. Indeed, we found that the exocytosis coefficient for infectious viral particles is larger than that for noninfectious L-particles (Figs. 2e, 4a), which may be due to differences in their envelope composition and internal contents^35,36^ (e.g., lacking capsid reduces the rigidity of L-particle envelope, leading to reduced membrane tension for the vesicle that releases an L-particle). The larger coefficient may facilitate the virion (rather than L-particle) to escape the host’s immune defense to infect new host for viral spread.

Besides exocytosis, membrane fusion also occurs during entry of enveloped virus^37^. Fusion of virus envelope with host cell membrane involves viral fusion proteins. In the case of viral fusion proteins of influenza virus^38^ and human immunodeficiency virus^39–41^, amphiphilic fusion peptides insert into host membrane to open fusion pore. In a study of herpesvirus entry^23^, more virus particles were captured at late stage of entry than at the beginning of fusion, suggesting that virus entry consists of a rapid fusion phase, followed by a slow entry phase. Such biphasic viral entry mirrors the distinctive release and recovery phases for vesicle exocytosis observed in this study (Supplementary Fig. S5). Therefore, the existence of distinctive release and recovery phases could be a conserved feature for both entry and exit of herpesviruses, and possibly even for other events involving membrane fusion.

## Materials and methods

### Virus production

PRV443, a PRV Becker strain containing EGFP fused to viral capsid protein VP26 (EGFP-VP26)^42^, was kindly donated by Prof. Lynn W. Enquist (Department of Molecular Biology and Princeton Neuroscience Institute, Princeton University, USA) and used for this study. The virus was prepared on BHK-21 cells and the viral titer was tested by plaque assay on BHK-21 cells. The virus titer was 6.6 × 10^7^ PFU/ml.

PRV86 (PRV Bartha EGFP-VP26) was constructed based on PRV Bartha strain by inserting the EGFP into the VP26 N-terminus between the second codon and the third codon, which could be used to analyze the viral particles. The detailed information is as follows. Firstly, the fragment containing UL34, UL35, and partial UL36 was amplified by PCR from PRV Bartha strain using the primer pairs 5′-GGGAGACCCAAGCTGGCTAGCATGGCGCGCGGCGGCGGCGGC-3′ and 5′-GGTTTAAACGGGCCCTCTAGACAGCCTGTGCAGCTGGAGGGA-3′. The PCR fragment was engineered into pcDNA3.1(+) with *Nhe*I and *Xba*I by using a ClonExpress II One Step Cloning Kit (Vazyme Biotech); the new clone was designated as pcDNA3.1-step1. Secondly, the three fragments, partial UL34, EGFP and UL35-partial UL36, were separately amplified using three primer pairs, 5′-GCGCCGAGCTGCGGCAGC-3′ and 5′-AGCTCCTCGCCCTTGCTCACGGACATGATGGCTCGGCGGGGA-3′ for partial UL34 (template pcDNA3.1-step1), 5′-GTGAGCAAGGGCGAGGAGCT-3′ and 5′-CTTGTACAGCTCGTCCATGC-3′ for EGFP (template pEGFP-N), and 5′-GCATGGACGAGCTGTACAAGTTCGACCCGAACAATCCCCG-3′ and 5′-TCGGGGGACTCGGGCCCAACGA-3′ for UL35-partial UL36 (template pcDNA3.1-step1). The PCR fragments were engineered into pcDNA3.1-step1 with *Afe*I and *Xcm*I by using a ClonExpress MultiS One Step Cloning Kit (Vazyme Biotech); the new clone was designated as pcDNA3.1-PRV86. Finally, the 5 μg of pcDNA3.1-PRV86 was transfected into BHK21 cells using lipofectamine 2000 (Invitrogen), then the PRV Bartha (MOI = 1) was added into the medium at 6 h post-transfection. Two days later, the medium was collected and the virus was purified by picking the positive plaque. The purified PRV86 was titered by performing plaque assay on BHK21 cells and the titer was calculated as 1 × 10^8^ PFU/ml.

### Primary hippocampus neuron culture and virus infection on EM grids

All animal procedures were performed following the guidelines of the Animal Experiments Committees at the University of Science and Technology of China. Primary cultures of dissociated hippocampal neurons were prepared as previously described with minor modifications^43,44^. Briefly, electron microscopic (EM) gold grids (Quantifoil R2/2 or R3.5/1 gold NH2 grids with 2 nm-thin-carbon film on top; Quantifoil Micro Tools GmbH) for culture were plasma cleaned with H_2_ and O_2_ for 10 s using plasma cleaning system (Gatan), exposed in UV light for 30 min, and coated with poly-l-lysine overnight. Coated grids were washed with distilled water and then Hank’s balanced salt solution (HBSS) for 24 h each. Hippocampi were harvested from embryonic, day 18 (E18) rats and were treated with trypsin at 37 °C for 15 min followed by washing and gentle trituration to dissociate the neurons. The dissociated cells were plated over the grids at a density of 40,000 cells/ml in 35 mm diameter Petri dish containing 1.5 ml neurobasal culture medium (Invitrogen) supplemented with 5% heat-inactivated bovine calf serum (HyClone) plus 5% heat-inactivated fetal bovine serum (PAA), 37.5 mM NaCl (Sigma), 1× Glutamax (Invitrogen), and 1× B27 (Invitrogen). After 24 h of incubation at 37 °C and 5% CO_2_, half of the medium was replaced by serum-free neurobasal culture medium (Invitrogen). At 6 DIV, neurons were infected with either PRV443 (PRV Becker EGFP-VP26) or PRV86 (PRV Bartha EGFP-VP26) at 10 MOI.

Cultured neurons infected by PRV443 at 14 h post infection (hpi) and by PRV86 at 24 hpi were plunge-frozen for cryoET and cryoCLEM, respectively. Briefly, immediately after being taken out from the incubator, the gold EM grids were placed in 37 °C extracellular solution (ECS, containing 150 mM NaCl, 3 mM KCl, 3 mM CaCl_2_, 2 mM MgCl_2_, 10 mM HEPES and 5 mM glucose (pH 7.3). Two microliters of ten times diluted 15 nm gold beads (CMC) was added on both sides of the grids. The grids were blotted and vitrified by plunge-freezing into liquid ethane by Vitrobot IV (FEI). Frozen-hydrated samples were transferred into liquid nitrogen for storage until use.

### Cryo correlative light and electron microscopy (cryoCLEM) imaging for PRV Bartha infected culture

Correlative imaging experiments were performed using the cryoCLEM system developed by Tao et al.^44^. In brief, a custom-built cryo-chamber with liquid nitrogen supply to fit on a light microscope (Olympus IX71 inverted fluorescence microscope) can accept an EM cryo-holder (Gatan 626 EM cryo-holder) through a side-port to position the EM grid above the objective lens of the light microscope. During experiments, the inside channel of the cryo-chamber was precooled and maintained at below −180 °C. Subsequently, the EM cryo-holder loaded with frozen-hydrated sample was inserted into the cryo-chamber for fluorescence imaging. For each field of view, two images were collected, one in bright-field and one in EGFP channel (Ex:470/40, DM: 495, Em: 525/50; Chroma, 49002), using a ×40 air objective lens (Olympus LUCPLFLN ×40, NA 0.6) and an ANDOR NEO sCMOS camera (Andor). Immediately after the light microscopy (LM) imaging, the EM cryo-holder with grid was directly transferred into an FEI Tecnai F20 transmission electron microscope for correlative cryoEM/cryoET imaging. The index of the finder grids and the patterned carbon holes on Quantifoil EM grids that can be visualized by both bright-field light microscopy and EM were used for the correlation between light and electron microscopy. Finally, tomographic slices were fine aligned and merged with the fluorescence images (using *Midas*^45^ and ImageJ^46^) to identify virus particles.

### Cryo electron tomography

CryoET data were collected using FEI Tecnai F20 transmission electron microscope equipped with 4K × 4K 4-ports readout CCD camera (Eagle, FEI) and operated at an acceleration voltage of 200 KV. To find the location of viral exocytosis, low magnification (×4400) cryoEM images with low electron dose (~0.1e^−^/Å^2^) were recorded for all areas of neurons where ice was thin enough for cryoEM imaging. These low-magnification images were used to identify candidate locations with viral particles. We then navigated to all the candidate positions and acquired high magnification (×14,500) images to identify locations of viral exocytosis. These locations were chosen to record tilt series. Tilt series were acquired in single-axis tilting from 0° to −48° and then from 3° to +48° at 3° interval, with a total accumulative electron dosage of about 90 e^−^/Å^2^ and a defocus value at −12 to −18 μm. Some of the images were acquired by tilting from 0° to −60° and then from 2° to +60° and appeared similar in quality with the rest of the data, consistent to previous reported observations^47^. Images were recorded at ×14,500 magnification with camera binned by 2 to make the final pixel size of 1.509 nm/pixel.

### CryoET reconstruction and visualization

For each image in the tilt series, “hot” pixels (pixels with abnormally large or small density values) were deleted by “ccderaser” in IMOD^45^. All images in a tilt series were first coarse-aligned by cross-correlation. After coarse alignment, typically 10–50 gold beads were picked manually in nontilted images using “3dmod” in IMOD^45^. Then, gold beads in all images of tilt series were tracked with “beadtrack” in IMOD^45^. The positions of gold beads were fitted into a specimen-movement mathematical model resulting in a series of predicted positions for each bead. The residual errors (distance between the actual and predicted position) were recorded to facilitate tracking and fixing of poorly modeled points. Gold beads in each image with residual error larger than three pixels were removed and the centers of the other gold beads were corrected by visual inspection with “3dmod” in IMOD^45^. All images in tilt series were then finely aligned with these gold beads. Three-dimensional reconstructions were maneuvered with IMOD^45^ using the simultaneous iterative reconstruction technique with ten iterations. A total of 133 tomograms from 10 grids from 10 different cultures were used for this study. Out of 133 tomograms, 96 tomograms containing viral exocytosis events were used for the quantification of virus and L-particle exocytosis from the various parts of the cells. Among 96 tomograms, 36 are axons, 43 dendrites, 15 cell body, and 2 filopodia. Segmentation and surface rendering of the tomogram was done by volume tracer and color zone in UCSF Chimera^48^.

### Computational analysis of virus exocytosis

The tomographic slices (4.5 nm in thickness) containing both widest release pore and the center of capsid were extracted from 3D tomographic reconstructions with the “Slicer” tool in IMOD^45^. On these slices, we then drew contour lines along the cytoplasmic leaflet of each vesicular membrane and the envelope of each viral particle using “3dmod”^45^, generating two 2D line profiles, an open one for the vesicular membrane and a closed one for the envelope of each particle being released. We define the line connecting the start and end points of the 2D line profiles of the vesicular membrane as baseline, representing the release pore. A total of ten parameters of the 2D profiles were measured as follows (see Fig. 2c): (1) Pore width: the length of the baseline. (2) Depth of release cavity: the maximum height of the vesicular membrane profile above the baseline. (3) The pore membrane angle: angles between all the profile line sections on the left and right vesicular membrane and the baseline were calculated, and the minimum angle for each half was averaged. (4) Membrane curvature: the radius resulting from a fitted circle to the upper half of the area of envelope or vesicular membrane profile using the least-squares solution of a circle’s equation was calculated. The reciprocal of the radius represents the membrane curvature. (5) Exposed envelope percentage: the area of the envelope not covered by protein density to the total area of the envelope. (6) Center of the envelope: position of each picked point on the envelope weighted by the mean distance to the two nearest segmented points was calculated. The average of the weighted position of the points was used as the center of the envelope. (7) Off-center distance of capsid: Off-center distance of capsid was calculated as the distance from the center of the capsid to the center of the envelope. Negative value of the distance is the center of capsid outwards from the center of the envelope. (8) Distance from center of capsid or envelope to release pore: we calculated the distance using the standard formula, where the negative value means the center is inward from the pore and the positive value means the center is outward from the pore. (9) Cleft width: we calculated the minimum distance from each point on the profile line of the coated area of the envelope to the profile line of the vesicular membrane. The mean value was calculated as cleft width. (10) Radius of the envelope: we calculated the sum of the distance between each point in the envelope profile line as envelope perimeter. The envelope perimeter divided by 2π was calculated as the radius of the envelope.

In order to calculate the radius of clathrin-coated pits (CCPs) and clathrin coats, we extracted central tomography slices of CCPs using “Slicer” tool in IMOD^45^. The CCP and clathrin coat profile were subsequently segmented by drawing contour lines using “3dmod”^45^. Then a circle was fitted to either CCP or clathrin coat profile. The radius of the fitted circle was taken as the radius of CCP or clathrin coat.

Relative elapsed time of virions or L-particles exocytosis was calculated as follows. Because pore membrane angle increases monotonically during exocytosis, the values of pore membrane angle for all virions or L-particles can be sorted in ascending order and indexed (starting from 1), such that virions or L-particles with lower indices were at the beginning of exocytosis. Each index (1 through 199 for virions and 1 through 102 for L-particles) divided by the total number of virions (i.e., 199) or L-particles (i.e., 102) thus represents the cumulative frequency of an exocytosis event exhibiting that specific pore membrane angle. Since the frequency of particles observed at an exocytosis stage reflects the elapsed time of a viral particle through that stage, this cumulative frequency was used to estimate the relative elapsed time from time 0 through that particular stage.

Hyperbola regression^49^ was used to characterize the observed biphasic behavior of exocytosis by fitting to the plot of relative elapsed time as a function of pore membrane angle. As illustrated in Supplementary Fig. S2a–d, the target hyperbola function can be described by the following formula:

$$y = \beta _0 + \beta _1t + \beta _2\sqrt {\left( {t - \alpha } \right)^2 + \,\gamma ^2},$$

where *y* is the pore membrane angle and *t* is the relative elapsed time. In our regression, *γ* was set to 0.01 to enable a sharp bend at the “hyperbolic vertex” (Supplementary Fig. S2a). Through this fitting, four parameters can be determined (*β*_0_, *β*_1_, *β*_2_, *α*). *α* is the “hyperbolic vertex” of the fitted curve (Supplementary Fig. S2b), representing the phase transition point. Slopes of the two asymptotes, representing the speed of pore membrane angle increases in two phases, are *β*_1_ – *β*_2_ and *β*_1_ + *β*_2_, respectively (Supplementary Fig. S2c, d).

Other exocytosis parameters, including exposed envelope percentage, distance from center of capsid or envelope to release pore, pore width, and depth of release cavity, are also biphasic functions of the relative elapsed time; thus these functions can be fitted using the above-mentioned hyperbola formula. In contrast to the fitting of pore membrane angle, *α* values used in these fittings are fixed and equal to the “hyperbolic vertex” of the pore membrane angle. Therefore, the fitting to these functions can determine three parameters (*β*_0_,*β*_1_,*β*_2_). Likewise, the time of initiation of membrane flattening was determined by fitting the scatter plots of either vesicular or envelope curvature as a function of relative elapsed time to the above-mentioned hyperbola formula. The resulting *α* value represents the initiation of membrane flattening for vesicular or envelope membrane.

## Supplementary information

SUPPLEMENTAL MATERIAL

Supplementary video 1

Supplementary video 2

## Data Availability

All datasets relative to this work are provided to readers upon request.

## Supplementary information

**Supplementary information** accompanies the paper at (10.1038/s41421-019-0134-6).
