# Supplementary material for: Biphasic exocytosis of herpesvirus from hippocampal neurons and mechanistic implication to membrane fusion
Source: Cell Discov. 2020 Jan 14;6:2. doi: 10.1038/s41421-019-0134-6 (PMC6957672; doi:10.1038/s41421-019-0134-6)
Supplement: Supplementary file 1 — SUPPLEMENTAL MATERIAL [file 41421_2019_134_MOESM1_ESM.docx]

**Supplementary Materials**

**Supplementary figures and legends**

**
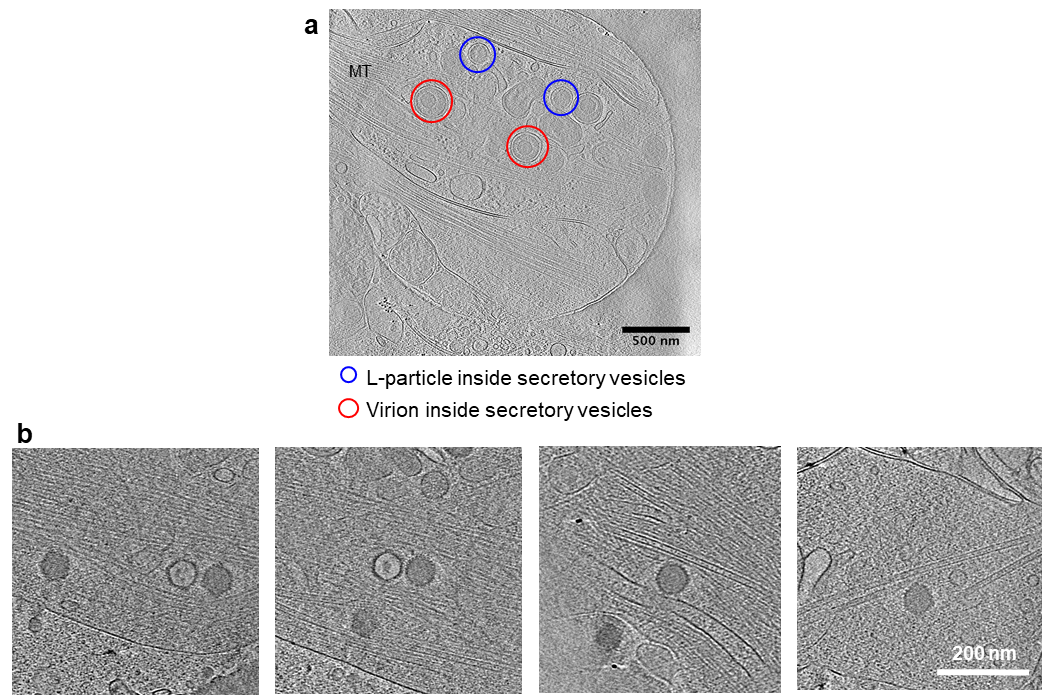
**

**Supplementary figure S1. PRV transport in hippocampal neuronal cells.**

**a**, 1.5nm-thick tomographic slice showing the transport of virus (red circle) and L-particle (blue circle) along microtubules (MT) in dendrite. **b**, Tomographic slices showing PRV Bartha capsids during retrograde transport.


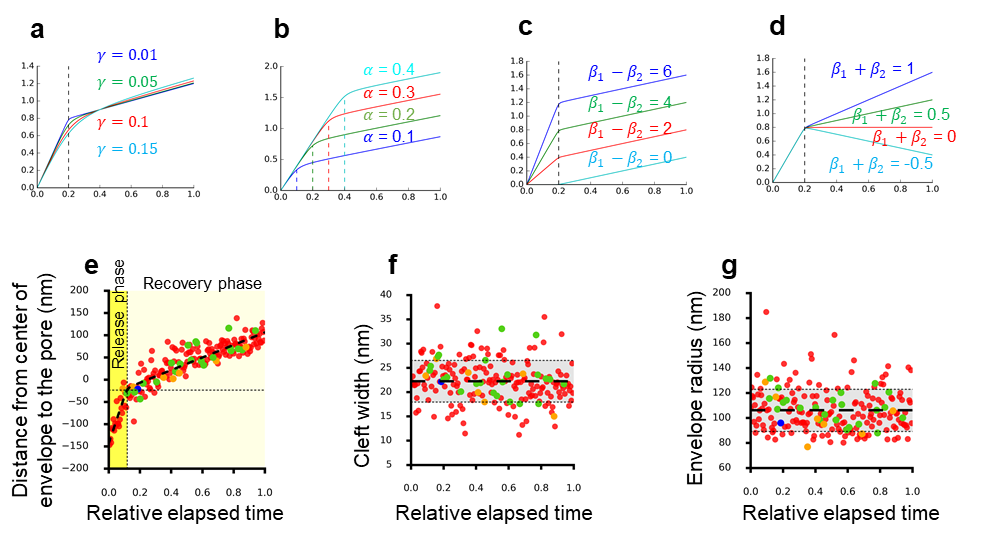


**Supplementary figure S2. Discovery of two phases of single-particle exocytosis of PRV.**
**a-d,** Four hyperbolas plotted with different sharpness $\gamma$ (**a**), *hyperbola vertex* position $\alpha$ (**b**), first slope $\beta_{1}-\beta_{2}$ (**c**), and the second slope $\beta_{1}+\beta_{2}$ (**d**). The dashed lines indicate *hyperbola vertex*. **e-g,** Scattered plots showing distance from center of envelope to the pore (**e**), cleft width (**f**), and envelope radius (**g**) plotted as a function of relative elapsed time of *single-particle exocytosis*. In (**e**), a hyperbola (black dashed lines) is fitted to the data points, and the vertex of this curve was used to define the point of transition separating the exocytosis into a release and a recovery phase (vertical dotted line). A horizontal dotted line is drawn from the vertex showing the value of the parameter at phase transition point. In (**f and g**), horizontal dashed line and grey area indicate the mean and standard deviation (SD), respectively. Dots are colored to differentiate the number of clathrin-coated pits (CCPs) invaginating from vesicular membrane during the *single-particle exocytosis*: one (green), two (yellow), four (blue), or without (red) CCPs.


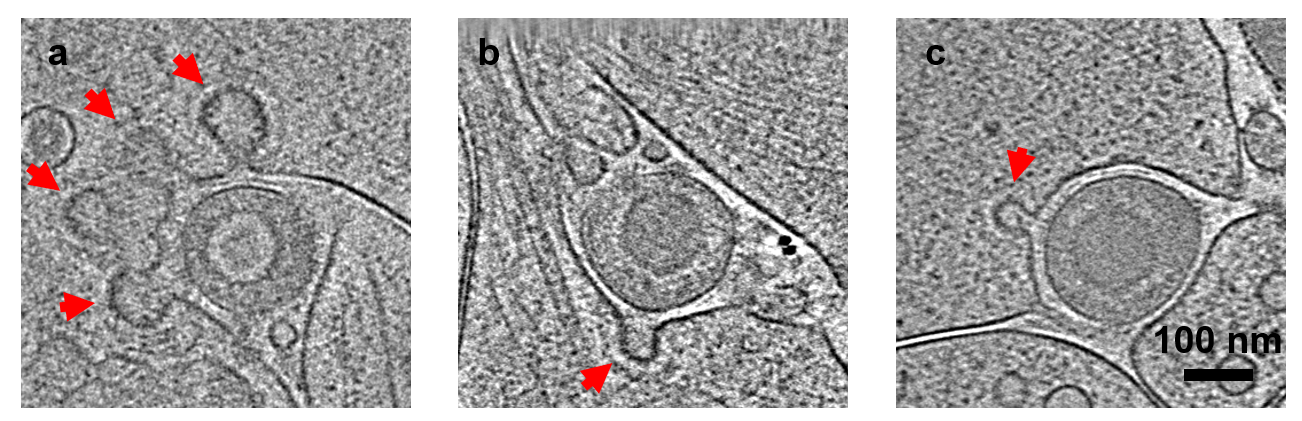


**Supplementary figure S3. Virus exocytosis involves clathrin-coated pit (CCP).**
**a-c,** Tomographic slices showing releasing viruses with CCP(s) (Red arrowhead) on cytosolic side of vesicular membrane.


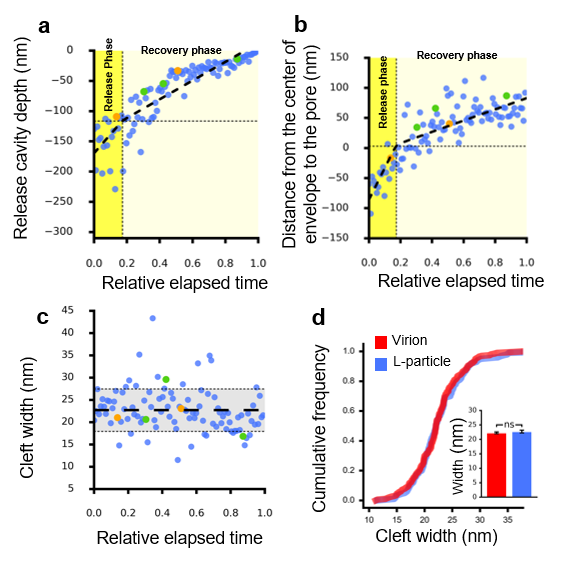


**Supplementary figure S4. Exocytosis of L-particle*.***
**a-c,** Release cavity depth (**a**), distance from center of envelope to the pore (**b**), and cleft width (**c**) plotted as the function of relative elapsed time of *single-particle exocytosis* of L-particle. In (**a and b**), a hyperbola (black dashed lines) is fitted to the data points. Vertical dotted lines in (**a and b**) show the point of transition separating the exocytosis into release and recovery phase. Horizontal dotted line shows value of each parameter at phase transition point. In (**c**), horizontal dashed line and grey area indicate the mean and standard deviation (SD), respectively. Dots are colored to differentiate the number of clathrin-coated pits (CCPs) invaginating from vesicular membrane during the exocytosis with one (green), two (yellow), or without (blue) CCPs. **d,** Cumulative frequency of viruses (red) and L-particles (blue) plotted as a function of their cleft width. Inset bar graph is the mean cleft width expressed as mean ± standard error of the mean (SEM); ns P>0.1 (two-tailed unpaired *t*-test).


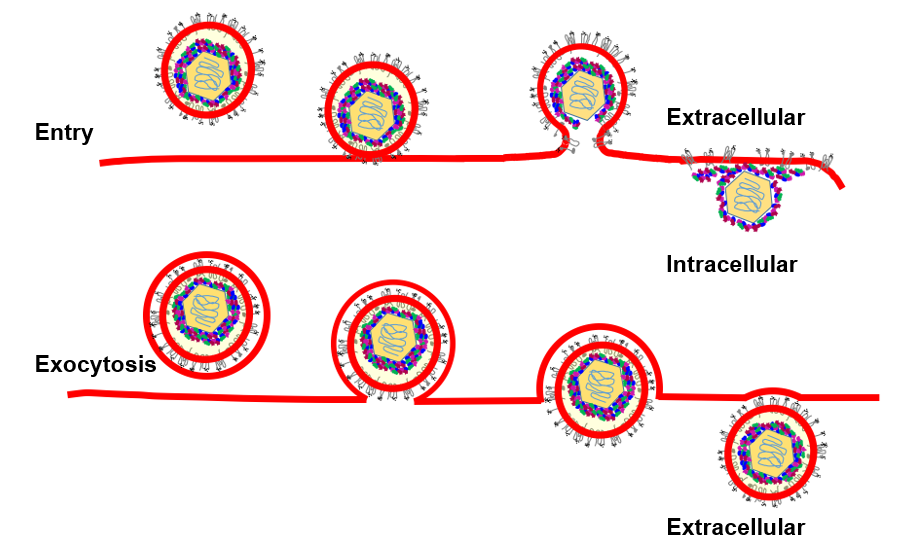


**Supplementary figure S5. Cartoon representation of PRV entry and exocytosis.**

**Supplementary movie titles**


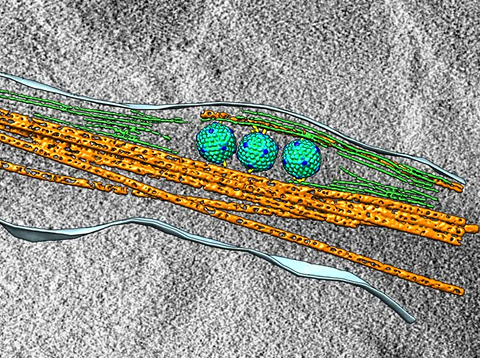


**Supplementary movie 1.** Slices through tomographic reconstruction of the PRV-Bartha capsid transportation in (Fig. 1e). Next, the rendering of the tomogram is shown and rotated. Capsids (cyan with blue pentons), microtubules (orange), actin (green) filaments, proteins density connecting capsid and cytoskeletons (yellow), and membrane (gray).


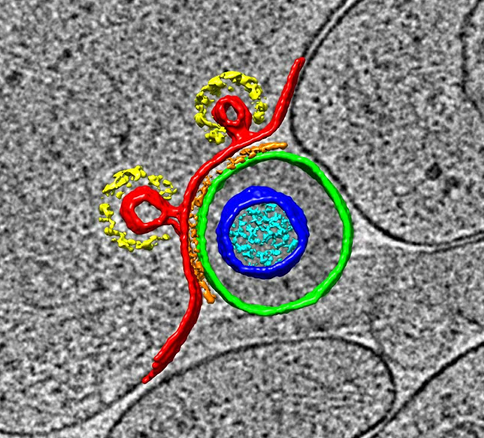


**Supplementary movie 2.** Slices through tomographic slices of the *single-particle exocytosis* with two CCPs in (Fig. 3). Together with 3D segmentation. Blue: capsid, cyan: dsDNA, green: envelope, orange: proteinaceous density layer between envelope and vesicular membrane, red: vesicular membrane, yellow: clathrin coat.
